# Vitamin D status and associated factors among HIV-infected children and adolescents on antiretroviral therapy in Kampala, Uganda

**DOI:** 10.1371/journal.pone.0253689

**Published:** 2021-06-24

**Authors:** Thereza Were Piloya, Sabrina Bakeera–Kitaka, Grace Paul Kisitu, Richard Idro, Sarah E. Cusick

**Affiliations:** 1 Department of Paediatrics, College of Health Sciences, Makerere University, Kampala, Uganda; 2 Baylor College of Medicine, Paediatric Centre of Excellence, Kampala, Uganda; 3 Department of Pediatrics, University of Minnesota, Minneapolis, Minnesota, United States of America; University of Sassari, ITALY

## Abstract

**Background:**

A high prevalence of suboptimal serum vitamin D has been reported among HIV infected children even in countries with high sunshine abundance throughout the year. Vitamin D is a potent immune modulator of innate and adaptive immune responses. Vitamin D regulates immune responses through the vitamin D receptor on CD4 cells. We aimed to determine the vitamin D status of HIV infected children and factors associated with suboptimal vitamin D.

**Methods:**

This was a cross sectional study. We enrolled children aged between 6 months and 12 years attending an outpatient paediatric HIV clinic. Serum 25-hydroxyvitamin D (25(OH)D) was measured using the electrochemoluminisence method. Suboptimal vitamin D was defined as 25(OH)D <30 ng/ml, vitamin D insufficiency and deficiency were 21–29 ng/ml and <20 ng/ml respectively. Anthropometry, physical exam and medical history were documented. Logistic regression was performed.

**Results:**

We enrolled 376 children with mean age (sd) 8.05 years (3.03), a median (IQR) duration of ART of 5.9 years (3.2–8.4). Majority of the children (64%) had been exposed to non nucleoside reverse transcriptase inhibitors (NNRTIs). A third were severely immunosuppressed (CD4% ≤15%) at ART initiation. At the time of the study, the majority (89%) were virologically suppressed (VL <1000 copies/ml). Prevalence of 25(OH)D <30 ng/ml was 49 (13%) of 375 participants and 11 (3%) had 25(OH)D <20 ng/ml. Lopinavir/ritonavir regimen was independently associated with 25(OH)D <30 ng/ml; OR 0.27 CI (0.13–0.57), p value-0.002. Serum 25(OH)D <20 ng/ml was associated with CD4 count ≤15% at ART initiation OR 6.55(1.30–32.9), p value—0.023 and use of NNRTIs; OR 10.9(1.22–96.2), p value—0.03.

**Conclusion:**

We found a low prevalence of suboptimal vitamin D compared to earlier reports. Severe immunosuppression at ART initiation and use of NNRTIs increases odds of deficiency. Vitamin D supplementation should be considered in severely immunosuppressed children initiating ART.

## Introduction

Human Immunodeficiency Virus (HIV) remains a burden in low-income countries despite the many interventions currently in place. In 2016, 2.1 million children under 15 years of age were living with HIV/AIDS globally, with two-thirds living in Sub Saharan Africa (SSA) [1]. Although Antiretroviral Therapy (ART) prolongs life and improves quality of life among HIV infected children [2], there is evidence of persistent inflammation and immune dysregulation in HIV infected individuals even with effective ART [3]. This inflammation coupled with cumulative drug toxicities predispose them to metabolic complications and bone diseases [3].

Vitamin D is a prohormone that has antinflammatory effects [4]. Vitamin D deficiency (VDD) is associated with greater inflammation by upregulation of inflammatory markers like, IL-6, TNF-α, activated monocyte phenotypes (CX3CR1+ and CCR2+) in HIV-infected patients [5], which have been related to tissue dysfunction, comorbidity development, AIDS progression, and death in HIV-infected individuals [6]. Severe VDD is associated with low CD4 counts and increased markers of inflammation in ART-naïve HIV-infected patients [7]. Additionally, high vitamin D levels have been associated with protection against the development of IRIS events [8] and decreased incidence of pulmonary tuberculosis and mortality among HIV-infected patients [9].

The main source of vitamin D is sunshine exposure. Children infected with HIV may be predisposed to VDD due to limited sun exposure as a result of ill health caused by opportunistic infections, concomitant treatment with ART and medications for opportunistic infections like ketoconazole which impair vitamin D metabolism [10] and also reduced dietary intake of vitamin D [11]. ART especially efavirenz may impair vitamin D metabolic pathways [12–14]. Long duration of ART is associated with vitamin D insufficiency (VDI) while a low CD4 count <200/μl, advanced stages of disease and current efavirenz use were independently associated with severe VDD [12]. Protease inhibitors (PI) use has shown no association with VDD [15]. VDD is also associated with opportunistic infections like tuberculosis and oral candidiasis as shown among HIV infected adults in a Tanzanian study [9].

The role of vitamin D as a potent immune modulator of innate and adaptive immune responses has been described [16–19]. Vitamin D regulates the immune responses through the vitamin D receptor (VDR) on CD4 cells. Vitamin D inhibits excessive production and action of T–helper 1 (innate immune system) thus prevents cellular inflammation [20, 21]. Furthermore, it plays a role in the innate immunity through activation of Toll-like receptors (TLRs) that leads to induction of the antimicrobial peptide cathelicidin and killing of intracellular bacteria [22]. Therefore VDD may affect the innate and adaptive immune response thus leading to disease progression [23].

The Endocrine Society defines sufficient vitamin D as a level of serum vitamin D (25(OH)D) ≥ 30–100 ng/ml while levels below 30ng/ml are suboptimal. The suboptimal level is further categorized into VDD and VDI [10]. VDD is defined as 25(OH)D <20 ng/ml,usually it manifests as bone disease [10]. VDI defines serum 25(OH)D between 20–29 ng/ml which are levels that may be associated with other disease outcomes [10]. A median serum level of 25(OH)D of 26 ng/ml is associated with reduced overall risk of cardiovascular mortality while in adolescents a level of 25(OH)D >26 ng/ml is associated with lower odds of elevated blood pressure and other diseases like T1DM [24]. High rates of suboptimal serum vitamin D in HIV-infected children have been reported in the range of 29–90% depending on the season, latitude, and patient ethnicity[25–27]. Observational studies suggest that vitamin D status may impact HIV disease severity [28–30]. A study in South Africa showed that a higher CD4 count had decreased odds of VDD among HIV infected children [31].

VDI is increasingly being reported even among healthy children in Uganda despite being located at the equator with an abundance of the sun [32–34]. This is possibly due to change in lifestyle amongst the general population. From our observation, children living within and around the capital city where majority of these studies have been conducted are increasingly being kept out of the sun because of more indoor activities at school, changes in housing with limited space for outdoor play and increased screen time due to urbanisation. Screening and supplementation for vitamin D are not routinely done in HIV infected children and adolescents in low-income settings despite the recommendation by the Endocrine Society that calls for screening for vitamin D status in HIV infected patients due to high risk of VDD [10]. However, there is no evidence for supplementation in an environment which is rich in sunshine throughout the year. This study was designed to assist in developing hypotheses for bigger studies to understand the role of vitamin D in HIV infection in a setting with an abundance of sunshine and a high burden of infectious diseases. Therefore our aim was to determine the vitamin D status among HIV infected children in Uganda and the factors associated with suboptimal vitamin D. The factors measured included clinical, immunological and laboratory markers.

## Methods

### Study design and participants

This was a cross-sectional study carried out between April 2019 and September 2019 at the Baylor HIV Paediatric Clinic, located at the Mulago National Referral Hospital Complex in Kampala the capital city of Uganda. Uganda is located at the Equator, Latitude 0° N with an average temperature of 26°C in Kampala. The study was carried out over 6 months’ duration consisting both the wet rainy season of April to June and the dry hot season from July to September. The sun is present throughout the year; however, in the rainy season majority of the children especially those younger children < 5 years remain indoors, over swaddled and rarely play in the sun. We enrolled HIV infected children attending the clinic aged between 6 months and 12 years, who were taking ART, and those whose parents or guardians provided written informed consent and assent for children ages ≥ 10years.

We excluded those who were ill (fever > 38.5 degrees Celsius, difficulty in breathing, altererd level of consciousness and / or in shock) on the day of enrollement, those who were already on vitamin D supplementation other than the multivitamin supplements given at the clinic and children who were currently on steroids for at least 2 weeks because of an independent association of chronic steroid use and low serum 25(OH)D [35]. Those with known chronic kidney disease, liver disease and heart disease were also excluded.

### Procedures

Systematic sampling was used as the children came into the clinic for triage. The study nurse approached every 10th child in the queue to assess eligibility.

A structured questionnaire was used to collect clinical data related to HIV illness from the eligible participants’ clinic chart. Clinical data collected included WHO clinical staging of HIV/AIDS [36], CD4 cell percentage and absolute count at the time of ART initiation. Additionally, we captured the current viral load on ART, the current ART regimen and duration on that ART as well as any other current medications. We also captured the previous number of admissions and any previous severe infections since ART initiation from the patient and the history of sun exposure.

Anthropometric measurements were taken, including the weight in kilograms and height/length in centimetres of the children. Height-for-age and weight-for-age Z-scores were calculated using the WHO reference charts [37]. Weight-for-age Z-score and height-for-age Z-score < -2 SD was considered as underweight and stunted respectively. Weight-for- height Z-score <-2 SD for children aged less than 5 years and BMI for age Z-score < -2 SD for those aged 5 years and above was considered as wasted. Physical examination was done to assess for overt features of rickets.

Laboratory testing: A venous blood sample of 5 mL was drawn into a plain (red top) blood collection tube from each child. Blood was centrifuged, and serum was collected and stored at -80°C. Serum vitamin D level was determined by electrochemiluminescence immunoassay using a technique called Elecsys vitamin D assay. This is an immunoassay supplied by Roche Diagnostics, Germany, which measures the vitamin D concentrations in the range of 4–100 ng/ml. Suboptimal 25(OH)D was defined as a level < 30ng/ml. VDI was defined as serum levels of 25(OH)D between 21–29 ng/ml and VDD was defined as serum levels of 25(OH)D < 20 ng/ml [10]. The parathyroid hormone (PTH) was measured using electrochemiluminescence immunoassay. Serum PTH level > 65 pg/ml was considered as hyperparathyroidism.

Serum calcium, phosphorus, alkaline phosphatase (ALP) were analyzed using a fully automated COBAS 6000 (Roche Diagnostics GmbH, Germany) machine. The calcium level of 8-10mg/dl (2–2.5 mmol) was considered normal, phosphorus: normal range infancy; 4.5–8.3 mg/dl (1.45–2.68 mmol), childhood; 3.7–5.6 mg/dl (1.19–1.8 mmol), alkaline phosphatase levels; was considered raised level at a value > 400 IU/dl. Viral suppression was considered at viral copies < 1000/ml as defined in the Uganda National HIV treatment guidelines [38].

### Ethics

Written informed consent was obtained from parents or guardians of all study participants. Written assent was obtained from all children aged 10 years and above. Ethical approval was granted by the institutional review board for human studies at Makerere University School of Biomedial Sciences (#SBS HDREC-618), Uganda National council of science and Technology (#HS294ES), Baylor College of Medicine, Houston, Texas (# H-45391) and University of Minnesota, Minneapolis, Minnesota (#STUDY00005173).

### Statistical considerations

Data entry and cleaning was done using Epidata version 3.1. Data was analyzed using statistical package, STATA Version 15.0. Patient characteristics were reported as frequency and percentage for categorical variables, mean and standard deviation (SD) for the normally distributed continuous variables with outliers and median and inter-quartile range (IQR) for continuous variables without outliers.

To determine the vitamin D status of the children; we got the proportion of the children with serum 25(OH)D <30 ng/ml from the total enrolled sample as suboptimal vitamin D level, we then further determined the proportion with categories of serum 25(OH)D <20 ng/ml, 21–29 ng/ml and ≥30 ng/ml as VDD, VDI and sufficient D level respectively.

To determine the factors associated with suboptimal vitamin D; our major outcome was defined as a dichotomized variable based on 25(OH)D < 30 ng/ml and > 30 ng/dl. Socio-demographic, clinical variables and biochemical variables were compared against the outcome using chi-square test or fisher’s exact when cell sizes were small (< 5 children). Multivariable analysis for factors independently associated with serum 25(OH)D <30 ng/ml were performed using a logistic regression model. Further analysis was done to determine variables that were associated with vitamin D level <20 ng/ml. A p-value <0.05 was considered statistically significant. The sample size of 376 was calculated using an assumed prevalence of vitamin D deficiency among HIV infected children of 57% from a Tanzanian study [39], with a margin of error of 5% and a standard normal value at confidence interval of 95% (Z_α2_ = 1.96).

## Results

We enrolled 376 particpants between April 2019 and September 2019.

**Fig 1:**
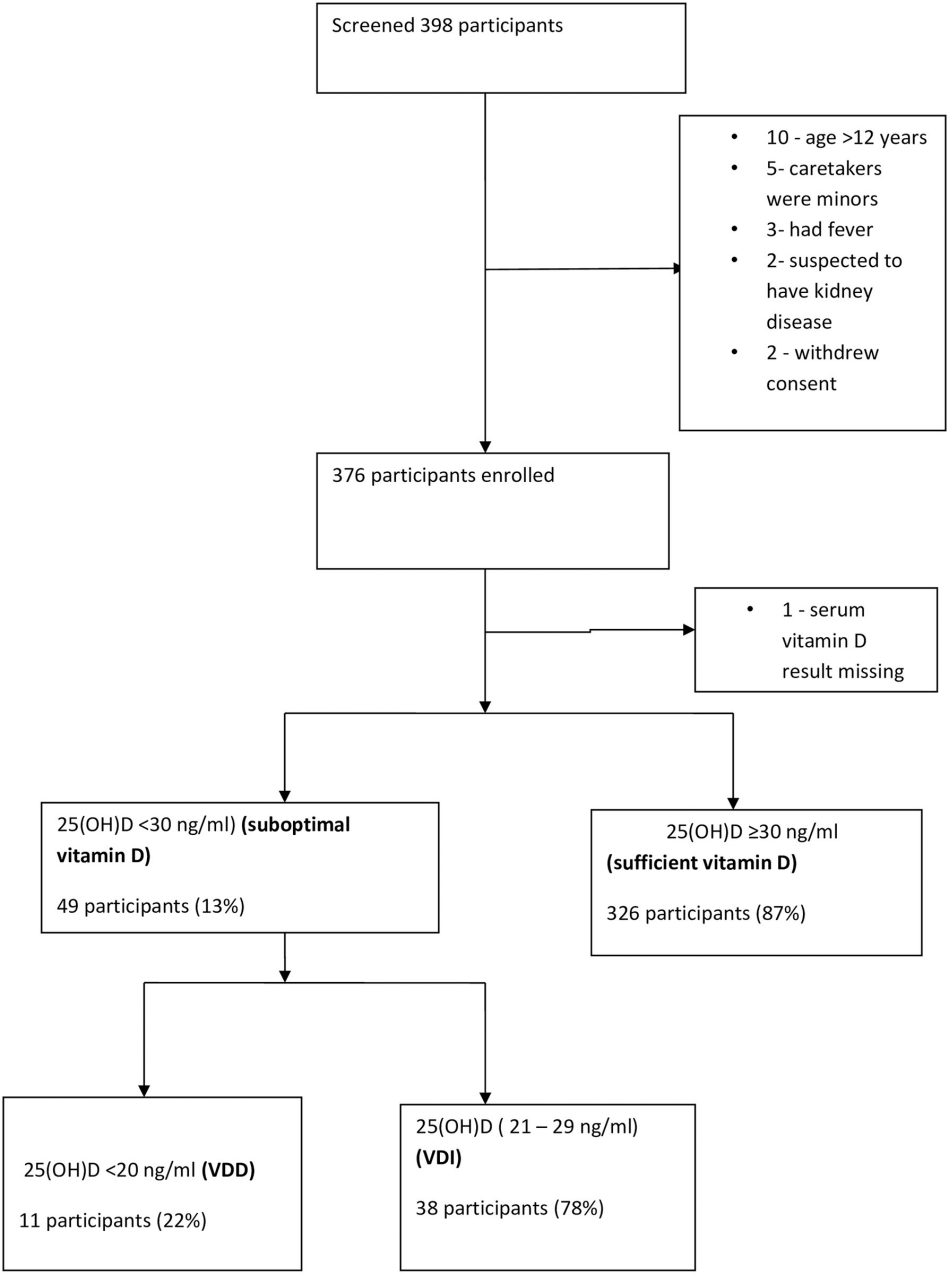
The study profile.

The study Profile is shown in Fig 1 below.

### Baseline characteristics

The mean age (SD) of the study participants was 8.05 years (3.03) with a median (IQR) duration of ART of 5.9 years (3.2–8.4). The median age (IQR) of ART initiation was 1.58 years (0.75–3.25). Majority of the children 64% (242 of 376 participants) had been exposed to non nucleoside reverse transcriptase inhibitors (NNRTIs); efavirenz and nevirapine. About 65 participants (17%) were underweight (weight for age zscore < -2) and more than a quarter (28.7%) of the participants were stunted (Table 1). Only 17 of the 376 participants (4.5%) had suffered from tuberculosis. Almost 99% of the study participants reported daily sun exposure.

**Table 1:** Sociodemographics and clinical characteristics.

Characteristic	25(OH)D ≥30 ng/ml, n (%)	25(OH)D <30 ng/ml, n (%)	P value
**Age (n = 375)**			
<5	64(92.8)	5(7.2)	
5–12 yrs	262(85.6)	44 (14.4)	0.120
**Gender (n = 375)**			
Male	150(84.7)	27(15.3)	
Female	176(88.9)	22(11.1)	0.236
**CD4%at initiation (n = 338)**			
> 15	194(87.8)	27(12.2)	
≤15	100(85.5)	17(14.5)	0.548
**Current Viral load (copies/ml) (n = 359)**			
≥ 1000	36(92.3)	3(7.7)	
<1000	274(85.6)	46(14.4)	0.260
**Height for age (n = 370)**			
≥-2 z score	231(88.5)	33(11.5)	
< -2 z score	91(85.8)	15(14.2)	0.669
**Weight for age (n = 374)**			
≥ -2 z score	269(82.8)	40(81.6)	
< -2 z score	56(17.2)	9(18.4)	0.845
**Weight for ht/ BMI for age Z-score (n = 370)**			
≥ -2 z-score	309(87.3)	45(12.7)	
< -2 z-score	13(81.3)	3(18.7)	0.486
**WHO stage at ART initiation (n = 375)**			
I& II	78(87.6)	11(12.4)	
III &IV	248(86.7)	38(13.3)	0.821
**Previous admissions (n = 371)**			
None	268(85.6)	45(14.4)	
Once	31(93.9)	2(6.1)	0.200
2–5 times	24(96)	1(4)	0.177
**First ART regimen(n = 370)**			
No	140(86.4)	22(13.6)	
Yes	183(88.0)	25(12.0)	0.655
*ART Regimen currently(n = 375)			
AZT/3TC/ EFV OR ABC/3TC/EFV OR TDF/3TC/	69(78.4)	19(21.6)	
AZT/3TC/ NVP OR ABC/3TC/NVP OR TDF/3TC/	38(90.5)	4(9.5)	0.101
AZT/3TC/ LPV/r OR ABC/3TC/LPV/r OR AZT	193(95.5)	9(4.5)	0.001
Dolutegravir/Others	31(72.0)	12(28.0)	0.426
**Duration for taking ART(n = 369)**			
< 6 years	168(88.9)	21(11.1)	
≥ 6 years	153(85.0)	27(15.0)	0.073
**Alkaline phosphatase (n = 371)**			
< = 400 iu	244(87.1)	36(12.9)	
>400 iu	78(85.7)	13(14.3)	0.727

AZT—zidovudine, 3TC—lamivudine, EFV- efavirenz, ABC-abacavir, NVP- nevirapine, TDF- Tenofovir, LPV/r- lopinavir/ritonavir.

About 2% (9 of 376 participants) had typical clinical signs of severe vitamin D deficiency including bone deformities such as bow legs, ‘knock- knees’ or rachitic rosary. Three participants had bow legs, 5 had ‘knock- knees’, 1 had a rachitic rosary. However, only one child with bow legs had 25(OH)D < 30ng/ml, all the rest of the 8 children had sufficient vitamin D level. All these children were on ART and were virologically suppressed. All the children with bow legs and rachitic rosary were stunted while only one child with ‘knock-knees’ was stunted. None of the children with bow legs was wasted while the one with rachitic rosary and two with ‘knock-knees’ were wasted.

The mean (SD) viral load (VL) of the participants at ART initiation was 531,226.1 (1542359) as compared to the current mean (SD) VL 15,871.1 (164030.6). Majority, 321 of 360 participants (89%) were virologically suppressed (VL <1000copies/ml) at the time of the study. Normocalcemia was detected in 305 participants (80%), none was hypocalcemic and 70 participants (18.7%) were hypercalcemic. Most of the participants 345 (92%), had normal parathyroid hormone while 282 participants (75%) had normal serum phosphorus and a quarter of participants (92) had low phosphorus.

### Vitamin D status of HIV infected children

Sufficient serum 25(OH)D ≥ 30 ng/ml was demonstrated in 326 participants (87%) while we found suboptimal level of serum 25(OH)D < 30 ng/ml in 49 participants (13%). Of those with suboptimal vitamin D; 11 of the 49 participants (22%) had 25(OH)D <20 ngml (VDD) while 38 participants (78%) had serum levels in range of 20–29 ng/ml (VDI). The median (IQR) 25(OH)D was 49.64 ng/ml (7.29–89.75).

### Factors associated with suboptimal vitamin D

Children on lopinavir/ritonavir regimen were less likely to have serum 25(OH)D <30 ng/ml; OR 0.27 CI (0.13–0.57), p value—0.001 (Table 2).

**Table 2:** Factors associated with suboptimal vitamin D (serum 25(OH)D <30 ng/ml) among HIV infected children.

Characteristic	Unadjusted Odds ratio, 95% CI	P value	Adjusted Odds ratio,95% CI	P value
**Age**				
<5	Ref		Ref	
5–12 yrs	2.14(0.82–5.64)	0.120	1.16(0.33–4.04)	0.814
**Gender**				
Male	Ref			
Female	0.69(0.38–1.27)	0.236		
**CD4%at initiation categorized**				
> 15	Ref		Ref	
≤15	1.22(0.64–2.35)	0.548	1.12(0.56–2.21)	0.748
**Current Viral load (copies/ml)**				
≥ 1000	Ref			
<1000	2.01(0.59–6.81)	0.260		
**Height for age**				
≥-2 z-score	Ref			
< -2 z-score	1.15(0.59–2.23)	0.669		
**Weight for age**				
≥ -2 z-score	Ref			
< -2 z-score	1.08(0.49–2.35)	0.845		
**Weight for ht/ BMI for age Z-score**				
≥ -2 z-score	Ref			
< -2 z-score	1.58(0.43–5.78)	0.486		
**WHO stage**				
I& II	Ref		Ref	
III &IV	1.09(0.53–2.22)	0.821	1.65(0.71–3.84)	0.243
**Previous admissions**				
None	Ref			
Once	0.38(0.09–1.67)	0.200		
2–5 times	0.25(0.03–1.88)	0.177		
**First ART regimen**				
No	Ref			
Yes	0.87(0.47–1.61)	0.655		
*ART Regimen currently				
AZT/3TC/ EFV OR ABC/3TC/EFV OR TDF/3TC/	Ref		Ref	
AZT/3TC/ NVP OR ABC/3TC/NVP OR TDF/3TC/	0.38(0.12–1.21)	0.101	0.35(0.10–1.18)	0.092
AZT/3TC/ LPV/r OR ABC/3TC/LPV/r	0.27(0.13–0.57)	0.001	0.25(0.11–0.59)	0.002
Dolutegravir/Others	1.41(0.61–3.25)	0.426	1.49(0.61–3.68)	0.381
**Duration for taking ART**				
< 6 years	Ref		Ref	
≥ 6 years	1.83(0.94–3.53)	0.073	1.21(0.57–2.56)	0.624
**Alkaline phosphatase**				
< = 400 iu	Ref			
>400 iu	1.12(0.57–2.23)	0.727		

AZT—zidovudine, 3TC—lamivudine, EFV- efavirenz, ABC-abacavir, NVP- nevirapine, TDF- Tenofovir, LPV/r- lopinavir/ritonavir.

**Fig 2:**
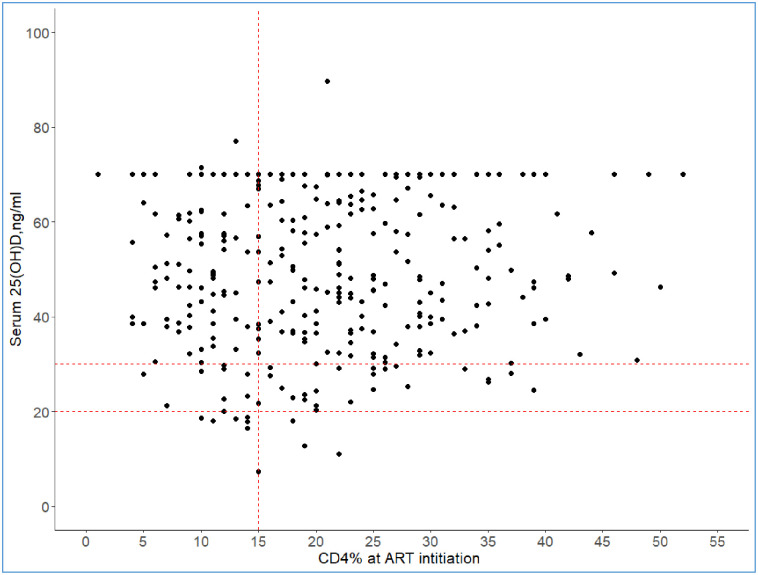
A graph of serum 25(OH)D against CD4% at ART initiation. The horizontal dotted lines show the 25(OH)D level at 20 ng/ml and at 30 ng/ml. The vertical doted line denotes the CD4% at 15% level. P value was calculated using chi square test comparing CD4% ≤15 and >15 with 25(OH)D <20 ng/ml and above.

However, it was noted that children with serum 25(OH)D <20 ng/ml (VDD) had low CD4 count (< 15%) at ART initiation OR 6.55(1.30–32.9), p value—0.023 as shown in Fig 2. They were also more likely to be on NNRTIs containing regimens with efavirenz and nevirapine OR 10.9(1.22–96.2), p value—0.03 as shown in Table 3. No biochemical markers like calcium, phosphorus, parathyroid hormone or alkaline phosphatase were independently associated with VDD.

**Table 3:** Factors associated with serum 25(OH)D <20 ng/ml (VDD) among HIV infected children.

Characteristic	25(OH)D > 20 ng/ml, N (%)	25(OH)D < 20 ng/ml, N (%)	Unadjusted Odds ratio, (95% CI)	P value	Adjusted odds ratio	P value
**Age**						
0–4 years	68 (18.7)	1(9.1)	Ref			
5–12 years	296 (81.3)	10(90.9)	2.3(0.29–18.3)	0.432		
**Gender**						
Male	169(46.4)	8(72.7)	Ref			
Female	195(53.6)	3(27.3)	0.33(0.08–1.24)	0.101		
**CD4%at initiation categorized**						
≤15	218(66.7)	3(27.3)	Ref		Ref	
>15	109(33.3)	8(72.7)	5.33(1.39–20.5)	0.015	6.55(1.30–32.9)	0.023
**Weight for age Z-score**						
≥ -2	301(82.9)	8(72.7)	Ref			
< -2	62(17.1)	3(27.3)	1.82(0.47–7.06)	0.386		
**BMI for age Z-score**						
≥-2	346 (96.1)	8(80.0)	Ref		Ref	
< -2	14(3.9)	2(20.0)	6.18(1.19–31.8)	0.029	4.91(0.64–37.7)	0.126
**WHO stage**						
I& II	88(24.2)	1(9.1)	Ref		Ref	
III &IV	276(75.8)	10(90.9)	3.19(0.40–25.3)	0.272	3.23(0.35–29.6)	0.298
**First ART regimen**						
No	161(44.7)	1(10.0)	Ref		Ref	
Yes	199(55.3)	9(90.0)	7.28(0.91–58.1)	0.061	4.98(0.56–44.1)	0.149
**Duration on ART**						
<6 years	182(50.8)	7(63.6)	Ref		Ref	
≥6 years	176(49.2)	4(36.4)	0.59(0.17–2.05)	0.408	0.41(0.08–2.00)	0.269
**Current Viral load (copies/ml)**						
≥1000	39(11.2)	0(0.0)	Ref			
<1000	309 (88.8)	11(100.0)	Cannot be calculated			
**Current ART Regimen**						
*LPV/r or PI based regimen	199 (54.7)	3 (27.3)	Ref		Ref	
NVP/EFV based regimen	165(45.3)	8(72.7)	3.22(0.84–12.3)	0.088	10.9(1.22–96.2)	0.032
**Alkaline phosphatase (n = 371)**						
< = 400 iu	276(76.7)	4(36.4)	Ref		Ref	
>400iu	84(23.3)	7(63.6)	5.75(1.64–20.12)	0.006	3.86(0.82–18.0)	0.085
**Serum calcium**						
2–2.5 mmol	295(81.3)	9(81.8)	Ref			
Above 2.5mmol	68(18.7)	2(18.2)	0.96(0.20–4.56)	0.963		

* AZT—zidovudine, 3TC—lamivudine, EFV- efavirenz, ABC-abacavir, NVP- nevirapine, TDF- Tenofovir LPV/r- lopinavir/ritonavir.

## Discussion

Our study found a low prevalence (13%) of suboptimal vitamin D (25(OH)D <30 ng/ml) as compared to previously reported studies among the HIV-infected children; with prevalence between 29–90% [25, 28, 31], and also among 135 healthy children aged <6 months the prevalence of 25(OH)D <20 ng/dl was 84% [40] while it was 48% among 95 healthy Ugandan children aged <7 years [32]. The major source of vitamin D is exposure to sunlight. Sunlight is abundant even in the rainy seasons in Uganda. Our study enrolments run across both the rainy season and dry season over 6 months. During the rainy season many children in our setting are kept indoors and are over swaddled especially for those aged <5 years. However, three quarters of our participants were enrolled in the dry months of June to September. We are uncertain whether the prevalence would be the same if this study was conducted in a purely rainy season and if the study population were of younger children aged <5 years.

Vitamin D deficiency has been neglected in many sunny regions despite increasing global reports of deficiency in these areas [41]. Therefore, there are few reports to compare our results with in the region regarding vitamin D status and HIV infected children. A study done in Tanzania found a high prevalence of insufficiency of 80% in HIV infected children [39], however, this study was conducted among infants thus making it difficult to compare with our study population with a mean age of 8 years. Also, the studies done among healthy children in Uganda were conducted among children of a young age group aged less than 8 years [33]. We believe the risk factors for vitamin D deficiency among the younger children aged < 5 years are plausibly different from those older [42, 43]. During a child’s infancy and preschool years, children tend to spend more time taking part in outdoor activities. As they grow, sedentary activities increase and adolescents tend to limit their outdoor activities because they prefer activities such as watching television or playing computer games. Additionally, their time outdoors is decreased because of prolonged study at school with outdoor physical activity usually limited to physical education at school. Also, dietary intake changes with age as the ingestion of unbalanced diets like fast and highly processed foods increases with age while the consumption of other nutritious foods containing vitamin D decreases [44].

This current study population comprised of older HIV infected children (mean age = 8years) and majority were healthy with virological suppression thus the children were more likely to be active and with good sun exposure thus the low prevalence.

Further still, the numbers of healthy children studied in previous reports in Uganda were too few to provide generalizable data to the population [33, 34]. Additionally, the lower prevalence may be accounted for by the different assay methods used for evaluating serum vitamin D by the various studies. This study used electrochemiluniscence assay while Cusick et al. [33] used chemiluminescent immunoassay and the high-performance liquid chromatography tandem Mass spectrometry was used by Sudfeld et al. in the Tanzanian study [39]. Multiple methodologies for 25(OH)D measurement exist but these are subject to variability due to inter assay differences in performance thus may explain the difference in results [45].

In this study we also found an association between severe immunosuppression at ART initiation and VDD. Other studies by Rutstein et al. [28] also found that decreased CD4 count was correlated to VDD and Mirza et al. [31] also found that a low CD4 count had an increased odds of vitamin D deficiency, although Kim et al. [46] found no association. Given the evidence demonstrating the role of vitamin D in innate and adaptive immunity, the association between low CD4 and VDD could be explained by the role played by the vitamin D and its receptor in activation and T-cell receptor signaling in naive T-cells [5], and it agrees well with data showing that VDD is associated with clinical progression [9] and lower CD4 counts even before initiation of ART [7], and with a poorer CD4 restoration on treatment [17]. However, we were unable to determine the association with the current CD4 count because the current treatment policy uses the viral load for ART monitoring and not the CD4 counts as previously [38].

The current HIV treatment guidelines in Uganda recommend initiation of ART at the earliest opportunity in all people with confirmed HIV infection, regardless of clinical stage or CD4 cell count [38]. This reduces the risk of delaying treatment and thus less likely to have many children starting ART with severe immunosuppression as compared to 7–10 years ago. This plausibly may explain the lower prevalence of VDD in this study, only 30% had started ART with CD4% <15. Therefore with this finding, it would be important to consider supplementation of all HIV infected children with severe immunosuppression with vitamin D because it’s an easy and cost effective intervention but also re-emphasize early initiation of ART for better outcomes.

We found no association between viral load and serum vitamin D although other studies have found that higher serum 25(OH)D is also associated with lower RNA viral load [46]. Majority (89%) of our study population were virologically suppressed with normal serum 25(OH)D. Vitamin D has been shown to increase autophagy, a process utilized by immune cells to kill intracellular pathogens [47]. Autophagy induced by physiological concentrations of 1,25(OH) vitamin D, leads to inhibition of HIV replication in HIV infected macrophages as demonstrated in vitro studies [48]. The children in our study had been receiving ART for a median duration of six years and many were healthy, therefore, the immunologic, HIV stage and other HIV related factors may have been normalized and could have compensated for the effects of the vitamin D status. We believe this could have been different in children who may have had advanced disease and may have just been initiated on ART.

Our study found an association between efavirenz use and VDD/VDI. Several studies have documented reduced vitamin D level in people with HIV treated with efavirenz [13, 27, 49]. Longitudinal studies demonstrate a 5 ng/mL decrease in 25(OH)D within 6–12 months after initiation of an efavirenz-based regimen [49] and an improvement on switching to darunavir based regimen which is a PI regimen [50]. The effect of efavirenz is hypothesized through the induction of cytochrome P450 enzyme, 24 hydroxylase which inactivates 25(OH)D and 1,25(OH)_2_D [49, 51].

We also found that lopinavir/ritonavir regimen was associated with lower odds of sub optimal vitamin D. This is consistent with other studies which report that ritonavir is associated with lower odds of low vitamin D [52], although some studies report no association [15]. In vitro, protease inhibitors have been shown to inhibit 25-hydroxylase and 1- α hydroxylase in a dose-dependent and reversible manner [53], resulting in decreased production of 1,25(OH)2D. Therefore it’s imperative to study the effect of various PIs on the vitamin D status.

### Limitations of this study

Our study had some limitations; we did not objectively assess the dietary and environmental effect (sun exposure) on the vitamin D status of our study participants. Additionally, we were unable to assess the effects of vitamin D deficiency/ insufficiency on bone due to financial constraints. We did not carry out any radiological tests for bone changes.

## Conclusions

In conclusion, we found a low prevalence of suboptimal vitamin D among stable virologically suppressed HIV infected children in Uganda. The use of lopinavir/ ritonavir was associated with reduced odds of suboptimal vitamin D, while efavirenz was associated with deficiency. We recommend that HIV infected children who are severely immunosuppressed or with advanced disease at ART initiation be supplemented with vitamin D.

## Supporting information

S1 Appendix Study questionnaire stamped. (DOCX)

S1 Dataset Vitamin D study complete data April 2020. (XLS)
