# Supplementary material for: Vitamin D status and associated factors among HIV-infected children and adolescents on antiretroviral therapy in Kampala, Uganda
Source: PLoS One. 2021 Jun 24;16(6):e0253689. doi: 10.1371/journal.pone.0253689 (PMC8224887; doi:10.1371/journal.pone.0253689)
Supplement: Supplementary file 1 [file pone.0253689.s001.docx]

Appendix V: Questionnaire

VITAMIN D STATUS; ASSOCIATED CLINICAL AND BIOCHEMICAL FACTORS AMONG CHILDREN AND ADOLESCENTS INFECTED WITH HIV AT BAYLOR PAEDIATRIC CLINIC, KAMPALA UGANDA

STUDY ID No……………………………

Date: ________________________________

SECTION A: Participant Demographics

1. Date of Birth DD/ MM/YR ……………………..
2. Age: …….. Years ………… Months
3. Sex:
   I. Male
   II. Female
4. Level of education
   I. Never went to school/ Not Applicable
   II. Primary
   III. Secondary
5. Religion
   I. Christian
   II. Muslim
   III. Others, specify ………………………………………

SECTION B: Caretaker Demographic information

6. Age (In years)……….
7. Sex:
   I. Male
   II. Female

MAKERERE UNIVERSITY
SCHOOL OF BIOMEDICAL SCIENCES
APPROVED
VALID UNTIL
17 OCT 2013
RESEARCH & ETHICS COMMITTEE
P. O. BOX 7072, KAMPALA

8. Who is the primary caretaker?

I. Mother

II. Father

III. Others; Specify ……………………………………

9. Level of education:

I. Never went to school

II. Primary level

III. Secondary level

IV. Tertiary level

10. Occupation

I. Unemployed

II. Peasant

III. Formal employment; Please Specify……………………………

IV. Informal employment

11. Who offers the main financial assistance in the household?

I. Father

II. Mother

III. Relative

IV. Sponsor

12. Level of income per month earned by the Next of Kin

I. Less than 100,000/=

II. 100000- 290000/=

III. 300000- 500000/=

IV. Above 500000/=

MAKERERE UNIVERSITY
SCHOOL OF BIOMEDICAL SCIENCES
APPROVED
VALID UNTIL
17 OCT 2019
RESEARCH & ETHICS COMMETTEE
P. O. BOX 7072, KAMPALA

SECTION C: Patient Information

13. Was the baby born at term or Preterm?

I. Term

II. Preterm

14. Is the child breastfeeding currently?

I. Yes (Go to Qn. 15)

II. No (Go to Qn.17)

15. Which type of breastfeeding is the child on?

I. Exclusive breastfeeding

II. Mixed feeding

16. Is the mother a vegetarian?

I. Yes

II. No

17. Is the child a vegetarian?

I. Yes

II. No

18. Does your child drink cow milk?

I. No

II. Yes (how many cups per week)---------------------

PAST MEDICAL HISTORY

19. What was the Age of ART Initiation? (LOOK IN MEDICAL CHART)

....

20. What is the Duration on ART

I. Less than 1 year

MAKERERE UNIVERSITY
SCHOOL OF BIOMEDICAL SCIENCES
APPROVED
VALID UNTIL
17 OCT 2013
64
RESEARCH & ETHICS COMMETTEE
P. O. BOX 7072, KAMPALA

II. 1 - 2 years

III. Above 2 years – 5 years

IV. Above 5 years

21. What ART Regimen is the child taking currently?

I. AZT/3TC/ EFV OR ABC/3TC/EFV OR TDF/3TC/EFV

II. AZT/3TC/ NVP OR ABC/3TC/NVP OR TDF/3TC/NVP

III. AZT/3TC/ LPV/r OR ABC/3TC/LPV/r OR AZT/3TC/ ATV/r

IV. AZT/3TC/ DRV/r

V. Others, specify…………………………………………………

22. Is this the 1st Regimen of ART your child is taking

I. Yes **(If Yes GO TO QUESTION 26)**

II. No **(If NO GO TO QUESTION 23)**

23. What Regimen was child taking Before?

I. AZT/3TC/ EFV OR ABC/3TC/EFV OR TDF/3TC/EFV

II. AZT/3TC/ NVP OR ABC/3TC/NVP OR TDF/3TC/NVP

III. AZT/3TC/ LPV/r OR ABC/3TC/LPV/r OR AZT/3TC/ ATV/r

IV. AZT/3TC/ DRV/r

V. Others, specify…………………………………………………

24. What was the duration on this previous Regimen?..................................

25. What was the reason for changing the ART regimen?

I. Treatment Failure

II. Drug Toxicities

III. New Opportunistic Infection Like TB

IV. Others, specify ………………………………………

26. Does your child have any current known chronic illness?

MAKERERE UNIVERSITY
SCHOOL OF BIOMEDICAL SCIENCES
APPROVED
VALID UNTIL
17 OCT 2019
RESEARCH & ETHICS COMMITTEE
P. O. BOX 7072, KAMPALA

I. Yes **(Go to number 27)**

II. No **(Go to number 28)**

27. Which chronic disease does the child have? (Tick as many)

I. Tuberculosis

II. Heart disease

III. Epilepsy

IV. Cerebral palsy

V. Bone diseases like fractures

VI. Cancer

VII. Others specify ……………..

28. How many times has the child been admitted since ART initiation?

I. None

II. Once

III. 2-5 times

IV. Greater than 5 times

29. What was the commonest cause of admission to the hospital? State two....

i) ………………………………………… ii)………………………………

30. Is the child on any medications other than the routine supplements and ART given at the clinic?

I. Yes **(Go to Qn. 31)**

II. No **(Go to Qn. 32)**

31. Which medications is the child on? Tick all that applies

I. Anticonvulsants

II. Antituberculous medications

III. Anticancer medications

MAKERERE UNIVERSITY
SCHOOL OF BIOMEDICAL SCIENCES
APPROVED
VALID UNTIL
17 OCT 2019
RESEARCH & ETHICS COMMETTEE
P. O. BOX 7072 KAMPALA

IV. Calcium supplements

V. Others, specify………………………………………

32. Any history of body aches/pains

I. Yes………………………………………..(Go to 33)

II. No…………………………………………..(Go to 35)

33. How long have the body aches been on?

I. < 2 weeks…………………….

II. 2-4 weeks……………………

III. > 4 weeks

34. Which sites are affected? (Tick all that applies)

I. Limbs

II. Trunk

III. Others

35. How often does your child get sun exposure?

I. Daily

II. 3 or more days a week

III. Less than 3 days a week

IV. Never

36. How many hours total a day:___________ hours

37. Which time of the day:

Morning _______hours

Noon _______hours

Afternoon _______hours

Physical Assessment:

38. Nutritional status:

a) Wasting Yes ……… No…………

b) Pedal Edema Yes ……… No…………

39. Anthropometric measurements

a) Weight (kg) ……………

b) Height (cm)……………………

c) MUAC (cm)…………………………

40. Physical signs of Rickets

a) Rachitic Rosary Yes………… No…………

MAKERERE UNIVERSITY
SCHOOL OF BIOMEDICAL SCIENCES
APPROVED
VALID UNTIL
17 OCT 2019
RESEARCH & ETHICS COMMETTEE
P. O. BOX 7072, KAMPALA

b) Bow legs Yes No

c) Knock Knees Yes………. No…………

d) Widened wrists Yes……….. No…………..

e) Other deformities , specify………………………………………..

Laboratory investigations:

41. VIRAL LOAD at initiation…………………..

42. VIRAL LOAD current………………………..

43. CD4 count at initiation………………………..

44. CD4 count current……………………………..

45. HB at initiation………………………………..

46. HB current……………………………………..

47. Serum electrolytes

a) Calcium ………

b) Phosphorus ……….

48. Liver function tests and Liver Enzymes

a) Serum Albumin

b) ALP …………..

49. Serum 25- Hydroxy Vitamin D ……….

50. PTH……………..

Person completing the Form INITIALS ……………………………

Date (dd/mm/yyyy) …………………………………

MAKERERE UNIVERSITY
SCHOOL OF BIOMEDICAL SCIENCES
APPROVED
VALID UNTIL
17 OCT 2019
RESEARCH & ETHICS COMMETTEE
P. O. BOX 7072, KAMPALA
